# On the Value of Urinary Analysis in the Diagnosis and Treatment of Hepatic Diseases

**Published:** 1865-02

**Authors:** George Harley

**Affiliations:** Professor in the University College, London.


*On the Value of Urinary Analysis in the Diagnosis and Treatment of Hepatic Disease.* By GEORGE HARLEY, M. D., Professor in University College, London.

In his paper before the British Medical Association, the author began by saying that, as the practice of medicine is simplified in direct proportion as our means of "physical diagnosis" increase, he was glad of having the opportunity of calling attention to the fact that a knowledge of the condition of the urine is of as great assistance in the diagnosis of affections of the liver as of those of the kidney. Hitherto, the only physical means we possed of detecting and distinguishing the various forms of hepatic disease did not extend beyond the knowledge of the position and size of the liver by percussion, the absence of bile from the stools by inspection, and the presence of biliary pigment in the urine by the application of nitric acid to that secretion. Everyone, however, must have met with cases of obscure hepatic disease where these means of research proved totally inadequate to their requirements. This circumstance has led several practitioners to seek for further aids to diagnosis in such cases; and consequently, at various times during the last few years, valuable suggestions have fallen from different members of the profession both here and abroad. For example, Dr. Eiselt, of Prague, has called attention to the fact that in cases of melanotic cancer of the liver, the true nature of the affection may be sometimes discovered during life by the presence of melanine in the patient's urine. Urine containing melanine, the author said, although of the normal color when first passed, gradually becomes of a dark hue, even as dark as porter, when left for some hours exposed to the air. This change appears to be the result of a slow oxidation of the pigment. In the second place, Frerichs, in his admirable treatise on Diseases of the Liver, states that two substances, tyrocine, and leucine, which were formerly only known to the scientific chemist, are invariably to be found in the urine of patients laboring under acute or yellow atrophy of the liver. Dr. Harley said he had been able to verify this statement in the urine of a young married woman who died from this most fatal form of disease. Dr. Wilkes, he stated, had brought the case under the notice of the Pathological Society, and a report of it would be found in the "Transactions." Dr. Harley mentioned an interesting case of chronic atrophy of the liver, the result of obstruction from disease of the pancreas, in the urine of which he found both tyrocine and leucine. He had seen the gentleman several times along with Mr. Prance, and they noticed that as the disease advanced the quantity of the abnormal ingredients increased. After death, crystals of tyrocine were found in the liver. Dr. Harly recommended that in all cases of obscure hepatic disease these substances should be looked for; and said that in the majority of cases they were readily detected in the concentrated urine by means of the microscope. The tyrosine appears as needles and little stars; the leucine as round yellow balls, some of which are occasionally spiculated.

The author next proceeded to direct attention to the method he had recently laid before the profession of distinguishing between jaundice arising from suppression and jaundice the result of obstruction—two forms of disease so ably described by Dr. Budd. As the treatment of jaundice from suppression ought to be very different from that adopted in jaundice from obstruction, it is of essential importance to be able to distinguish the one from the other. After alluding to the great differences of opinion that have hitherto existed regarding the presence of the biliary acids in the renal secretions in cases of hepatic disease, the author pointed out how the discrepancies arose from the fact that sufficient attention had not been paid to the kind of jaundice under which the patient labored. He said he believed that when bile acids occurred in the urine in any quantity, their presence might be regarded as a certain sign of the existence of some obstruction in the course or at the termination of the common bile duct. Dr. Harley then proceeded to demonstrate, by experiment, how easy it is to detect the bile acids in the urine by means of strong sulphuric acid and a small piece of white sugar. The sulphuric acid was so added as not to mix with the urine; the sugar floated at their line of contact, and after some minutes assumed a purple hue. In the urine containing no bile acid the sugar was simply browned.

Dr. Thudichum said the author had ascribed the doctrine he had propounded to Professor Frerichs. He, however, only watched the patients clinically; it was another physiologist who made the examinations, and discovered tyrocine and leucine. The disease in question was dogmatically termed atrophy, but he (Dr. Thudichum) as dogmatically objected to that term, for he had seen cases where the liver was certainly enlarged about one-third its ordinary size; and in one case, brought under his notice by Dr. Richardson, it was doubled in size. In some cases, so far from the liver being atrophied, it was hypertrophied. An excellent test was to put a drachm of the solution of the nitrate of mercury to the urine; the urine being then boiled, the white precipitate produced would be transformed into a dark purple color, and the solution itself would assume a partly purple color.

